# Anti-tumor efficacy of CKD-516 in combination with radiation in xenograft mouse model of lung squamous cell carcinoma

**DOI:** 10.1186/s12885-020-07566-x

**Published:** 2020-11-03

**Authors:** Min-Young Kim, Jung-Young Shin, Jeong-Oh Kim, Kyoung-Hwa Son, Yeon Sil Kim, Chan Kwon Jung, Jin-Hyoung Kang

**Affiliations:** 1 Laboratory of Medical Oncology, Cancer Research Institute, The Catholic University of Korea, Seoul, Republic of Korea; 2 Department of Biomedicine & Health Sciences, The Catholic University of Korea, Seoul, Republic of Korea; 3 Department of Radiation Oncology, Seoul St. Mary’s Hospital, The Catholic University of Korea, Seoul, Republic of Korea; 4 Department of Pathology, Seoul St. Mary’s Hospital, The Catholic University of Korea, Seoul, Republic of Korea; 5 Department of Medical Oncology, Seoul St. Mary’s Hospital, The Catholic University of Korea, 222, Banpo-daero, Seocho-gu, Seoul, 06591 Republic of Korea

**Keywords:** Irradiation, Vascular disrupting agent, Tumor necrosis, Tumor hypoxia, Squamous cell carcinoma of lung, Xenograft model

## Abstract

**Background:**

Hypoxic tumors are known to be highly resistant to radiotherapy and cause poor prognosis in non-small cell lung cancer (NSCLC) patients. CKD-516, a novel vascular disrupting agent (VDA), mainly affects blood vessels in the central area of the tumor and blocks tubulin polymerization, thereby destroying the aberrant tumor vasculature with a rapid decrease in blood, resulting in rapid tumor cell death. Therefore, we evaluated the anti-tumor efficacy of CKD-516 in combination with irradiation (IR) and examined tumor necrosis, delayed tumor growth, and expression of proteins involved in hypoxia and angiogenesis in this study.

**Methods:**

A xenograft mouse model of lung squamous cell carcinoma was established, and the tumor was exposed to IR 5 days per week. CKD-516 was administered with two treatment schedules (day 1 or days 1 and 5) 1 h after IR. After treatment, tumor tissues were stained with hematoxylin and eosin, and pimonidazole. HIF-1α, Glut-1, VEGF, CD31, and Ki-67 expression levels were evaluated using immunohistochemical staining.

**Results:**

Short-term treatment with IR alone and CKD-516 + IR (d1) significantly reduced tumor volume (*p* = 0.006 and *p* = 0.048, respectively). Treatment with CKD-516 + IR (d1 and d1, 5) resulted in a marked reduction in the number of blood vessels (*p* < 0.005). More specifically, CKD-516 + IR (d1) caused the most extensive tumor necrosis, which resulted in a significantly large hypoxic area (*p* = 0.02) and decreased HIF-1α, Glut-1, VEGF, and Ki-67 expression. Long-term administration of CKD-516 + IR reduced tumor volume and delayed tumor growth. This combination also greatly reduced the number of blood vessels (*p* = 0.0006) and significantly enhanced tumor necrosis (*p* = 0.004). CKD-516 + IR significantly increased HIF-1α expression (*p* = 0.0047), but significantly reduced VEGF expression (*p* = 0.0046).

**Conclusions:**

Taken together, our data show that when used in combination, CKD-516 and IR can significantly enhance anti-tumor efficacy compared to monotherapy in lung cancer xenograft mice.

**Supplementary Information:**

The online version contains supplementary material available at 10.1186/s12885-020-07566-x.

## Background

Lung cancer is one of the most common malignancies in both men and women, and is the major cause of cancer-related deaths worldwide [1]. Lung cancer is histologically classified as small cell lung cancer or non-small cell lung cancer (NSCLC). The latter accounts for approximately 85% of all lung cancers [2, 3]. Squamous cell carcinoma (SqCC), which accounts for approximately 30% of NSCLCs, still has a poor prognosis owing to limited treatment options [4].

Concomitant chemotherapy combined with radiation has traditionally been regarded as the standard treatment for locally advanced stage III NSCLC [5]. However, the 3-year survival rate is less than 15% [6] owing to severe toxicities caused by multi-modality treatment and frequent locoregional recurrence and/or distant metastases even after successful completion of treatment. Accordingly, there is an urgent need to develop new treatment strategies that not only enhance local effects, but also minimize side effects when anticancer drugs are simultaneously or sequentially combined with radiation.

In contrast to normal cells that can rapidly recover in response to radiation, cancer cells are more sensitive to radiation and, therefore are more likely to be killed by radiation. Radiation is effective for local antitumor control, and has been applied to a variety of solid tumors, including lung cancer, head and neck cancer, and cervical cancer. However, hypoxic or acidic areas within cancer tissues are known to be highly resistant to radiation. Hypoxia is known to cause recurrence, particularly in NSCLC patients with poor prognosis [7].

Unlike normal tissues, blood vessels within tumor tissues are formed in complex structures with abnormal shapes. Such abnormal vascular structures can become hypoxic, leading to an increased expression of hypoxia-inducible factor 1-alpha (HIF-1α). Increased HIF-1α induces angiogenesis by increasing vascular endothelial growth factor (VEGF) expression [8, 9]. Ultimately, a series of these events give rise to local progression and distant metastases through newly created blood vessels. In fact, about 50% of cancer patients receiving radiation become resistant to the treatment over time, with low oxygen tension in tumor tissues being the leading cause of local treatment failure [10].

To date, many studies on various forms of angiogenesis inhibitors have been carried out to tackle radiation resistance by effectively suppressing hypoxia-induced tumor angiogenesis. Vascular disrupting agents (VDAs) bind tubulin, thereby targeting existing blood vessels in the center of the tumor. Generally, these are classified as flavonoid and tubulin polymerization inhibitor VDAs. Flavonoid VDAs accentuate pathologic signaling by cytokines such as tumor necrosis factor (TNF) and VEGF, leading to changes in the actin cytoskeleton, increased vascular permeability, and endothelial apoptosis [11]. In contrast, tubulin polymerization inhibitors can disrupt the tubulin network of the cytoskeleton in endothelial cells, influence endothelial cell junctions, influence the actin cytoskeleton, and change the vascular shape, resulting in increased vascular permeability [11].

Some preclinical and clinical studies have reported the efficacy and safety profiles of VDAs [12–16]. In theory, radiation therapy is not effective at locally controlling hypoxic areas in tumor tissues. Unlike cytotoxic anti-cancer drugs or other angiogenesis inhibitors, VDAs mainly affect blood vessels located in the central area of the tumor. Therefore, combined treatment with VDAs and radiation may compensate for the limited effect that radiation alone has in the center of tumors.

CKD-516, a novel tubulin polymerization inhibitor, can selectively bind to tubulin in the endothelial cells of tumor vessels and block tubulin polymerization, thereby destroying the aberrant tumor vasculature [17]. This intracellular process can lead to a rapid decrease in blood flow and nutrient supply, resulting in rapid tumor cell death.

In this study, we investigated the anticancer efficacy of treatment with CKD-516 alone or in combination with low-dose radiation in short- and long-term administration schedules in an SqCC xenograft mouse model. Additionally, we investigated the expression of oncogenic signaling proteins involved in tumor hypoxia and angiogenesis.

## Methods

### Cell culture and reagents

The NCI-H520 (male, human squamous cell lung carcinoma) cell line (Cat. #HTB-182) was purchased from the American Type Culture Collection (Manassas, USA). Cells were cultured in RPMI-1640 medium (Welgene, Korea) supplemented with 10% (v/v) fetal bovine serum (FBS), 200 U/mL penicillin, and 200 μg/mL streptomycin (Gibco, Korea). Cells were maintained at 37 °C in a 5% CO_2_ incubator and subjected to mycoplasma contamination test using PCR Mycoplasma Detection Set (Cat. #6601, TaKaRa, Korea) before each experiment. Cells were authenticated by ATCC STR profiling service and ethics approval was not required for the cells used in this study. The potent tubulin polymerization inhibitor, CKD-516, was obtained from the Chong Kun Dang Research Institute (Korea). Working concentrations were freshly prepared in 1 × phosphate-buffered saline (PBS).

### Animals and xenograft models

BALB/c nude mice were used in this study because they are a suitable animal model for evaluating anti-cancer efficacy [18]. Four-week-old male BALB/c nude mice, with an average weight of 20 g, were purchased from Orient Bio (Seoul, Korea) and maintained under specific pathogen-free conditions. The mice were housed at 22.5 ± 0.2 °C with 50 ± 10% humidity in a 12 h light-dark cycle. Mice were fed with a gamma ray sterilized diet (TD 2018S, Harlan Laboratories Inc., America) and given autoclaved reverse osmosis (R/O) water and Aspen bedding (PG-3, LAS bedding, Germany). H520 cells (2 × 10^6^) were suspended in 100 μ L of serum-free RPMI-1640 medium, and injected subcutaneously into the right forearm of the mice. A total of 88 mice were used for this experiment. At the end of the experiment, mice were euthanized in a chamber by gradually increasing the concentration of CO_2_ gas. All surgical interventions and pre- and postsurgical animal care were carried out in accordance with the Laboratory Animals Welfare Act, the Guide for the Care and Use of Laboratory Animals, and the Guidelines and Policies for Rodent Survival Surgery provided by the Institutional Animal Care and Use Committee (IACUC) at the School of Medicine at The Catholic University of Korea (Approval number: CUMS-2015-0143-01). The IACUC and Department of Laboratory Animal (DOLA) at the Catholic University of Korea, Songeui Campus, was accredited by the Korea Excellence Animal Laboratory Facility from the Korea Food and Drug Administration in 2017 and acquired AAALAC International full accreditation in 2018.

### Drug treatment and irradiation

Mice were randomized into control and treatment groups. When the tumor volume in mice reached 600–800 mm^3^ in diameter, mice were divided into seven groups (6–10 mice per group): 1) vehicle, injected with PBS weekly; 2) CKD-516, 3 mg/kg; 3) CKD-516, 5 mg/kg; 4) irradiation (IR), 2 Gy/day; 5) IR, 4 Gy/day; 6) CKD-516 + IR (day 1); 7) CKD-516 + IR (days 1 and 5) (Additional file 1). IR was performed daily for 5 days, and then mice were rested for 2 days per week. CKD-516 was administered with two treatment schedules on day 1 (d1) or days 1 and 5 (d1, 5) 1 h after IR. Groups 1, 2, 5, 6, and 7 received short-term treatment, while groups 1, 2, 4, and 6 received long-term treatment. Mice were irradiated with a Cs-137 Gammacell 3000 Elan Irradiator (MDS Nordion, Canada) at a dose rate of 5 Gy/min (1450 Ci). The energy of the Cs-137 source was 0.662 MeV. Mice were anesthetized before irradiation at 22 °C. The immobilization device is shown in Additional file 2A. A 4 mm thickness lead shield was designed, in which a 50 mL tube can be inserted. The mouse was placed into the tube and successfully immobilized during IR. IR toxicity was monitored based on body weight. Tumor volume was measured every other day using calipers throughout the experimental period. Tumor volume was calculated based on the following formula: tumor volume [mm^3^] = {(length [mm]) × (width [mm])^2^}/2.

### Measurement of vascular perfusion in tumors

At the end of the drug treatment schedule, 10 mg/kg Hoechst 33342 solution (Cat. #B2261, Sigma-Aldrich, USA) was injected into the tail vein to measure vascular perfusion within the tumor. Mice were euthanized 1 min later. Frozen tissues were prepared using an optimal cutting temperature compound. Hoechst 33342 fluorescent images (7 μm sections) were captured by fluorescence microscopy (Axiovert 200, Zeiss, Germany) with OLYPUS cellSens Standard version 1.6.

### Analysis of hypoxic tumor area

The Hypoxyprobe™-1 Plus Kit (Cat. #HP2–1000, Hypoxyprobe, USA) was used to evaluate the area of hypoxia within tumor tissues. Paraffin-embedded tissue sections (4 μm in thickness) were deparaffinized with xylene. Endogenous peroxidase activity was blocked by immersing the sections in methanol with 3% (v/v) hydrogen peroxide for 5 min, followed by washing with water and 1× tris buffered saline (TBS) (Cat. #IBS-BT005–1, iNtRON, Korea) supplemented with 0.1% (v/v) Tween 20 (Cat. #T1072, Biosesang, Korea) (TBST). Antigen retrieval was performed by boiling the sections in citrate buffer pH 6.0 (Cat. #CBB500, ScyTek Laboratories, USA). To block nonspecific binding, sections were incubated with a protein blocking agent, 1% (w/v) bovine serum albumin (Cat. #BSA025, BOVOGEN, Australia) for 5 min and washed with 1× TBST buffer. Sections were then incubated with FITC-MoAb1 (primary MoAb; 1:100, included in the kit) for 30 min at 23 °C. After washing with 1 × TBST 3 times, sections were incubated with HPR-conjugated rabbit anti-FITC (included in the kit) for 30 min at 23 °C. After washing with 1 × TBST 3 times, peroxidase activity was tested using 3,3′- diaminobenzidine (DAB) (Cat. #D41–125, GBI Labs, USA) and sections were counterstained with hematoxylin. The hypoxic area was scored on the whole stitched images as the positively stained area with pimonidazole relative to the entire tissue area using the Pannoramic MIDI slide scanner (3DHISTECH Ltd., Hungary) equipped with the Pannoramic Viewer software version 1.15.3. At least four whole sections from each tumor were examined. The staining intensity of tumor cells was graded as follows: 0, absent; 1, weak (light brown); 2, moderate (brown); and 3, strong (dark brown). We considered grades 2 and 3 as positive for pimonidazole staining and analyzed the hypoxic area by calculating the percentage of the positive area of the entire tumor tissue.

### Assessment of tumor necrosis

Hematoxylin and eosin (H&E) stained sections were imaged with a Pannoramic MIDI Slide Scanner (3DHISTECH Ltd.) to analyze the area of tumor necrosis. At least four whole sections from each tumor were evaluated. H&E staining was performed according to the manufacturer’s instructions.

### Immunohistochemical staining

Paraffin-embedded tissue sections (4 μm in thickness) were deparaffinized with xylene (Cat. #X0097, SAMCHUN, Korea). Endogenous peroxidase activity was blocked by immersing sections in methanol with 3% (v/v) hydrogen peroxide for 10 min, followed by washing with water and PBS. Antigen retrieval was then performed by boiling the sections in citrate buffer. Sections were incubated with Ki-67 (Cat. #4203–1, EPITOMICS, UK), Glut-1 (Cat. #2944–1, EPITOMICS,), HIF-1α (Cat. #20960–1-AP, Protein Tech, USA), CD31 (Cat. #ab28364, Abcam, UK), and VEGF (Cat. #SC-7269, Santa Cruz, USA) antibodies at 4 °C overnight, at a 1:100 dilution. Slides were washed with PBS and then incubated with a biotinylated secondary antibody provided in the Polink-2 Plus HRP Detection Kit (Cat. #D41–125, GBI Labs, USA) for mouse and rabbit antibodies with DAB Chromogen (included in kit) for 10 min at 23 °C. After washing with water and PBS, the peroxidase activity was tested with DAB, and the sections were counterstained with hematoxylin. Ki-67, Glut-1, HIF-1α, CD31, and VEGF expression levels were scored based on the staining intensity of tumor cells and the relative proportion of positively stained cells among total tumor cells using the Pannoramic MIDI Slide Scanner (3DHISTECH Ltd.) equipped with the Pannoramic Viewer software version 1.15.3. At least four whole sections from each tumor were evaluated. The slide sections were interpreted by a board-certified pathologist. The staining intensity of tumor cells was graded as follows: 0, absent; 1, weak (light brown); 2, moderate (brown); and 3, strong (dark brown). We considered grades 2 and 3 as positive for antibody staining and calculated the percentage of the positively stained area of total tumor tissue.

### Statistical analysis

The results obtained from at least 6 mice in each experiment are presented as the mean ± the standard deviation or median ± interquartile range. All data were tested using the Kolmogorov-Smirnov and Shapiro-Wilk tests for normality. Student’s t-test and Mann-Whitney U test were used to determine the statistical significance of the two different groups. The comparison between the control and treatment groups was analyzed using two-way analysis of variance (ANOVA) followed by Scheffe’s post-test. A *p* value of < 0.05 was considered statistically significant, and a *p* value of < 0.01 was considered to be highly statistically significant. All data were analyzed using Microsoft Excel 2010 for Windows 7 (Microsoft, Seoul, Korea) and PASW Statistics version 18.0 (SPSS Inc., Chicago, IL, USA).

## Results

### Anti-tumor efficacy of CKD-516

**Fig. 1:**
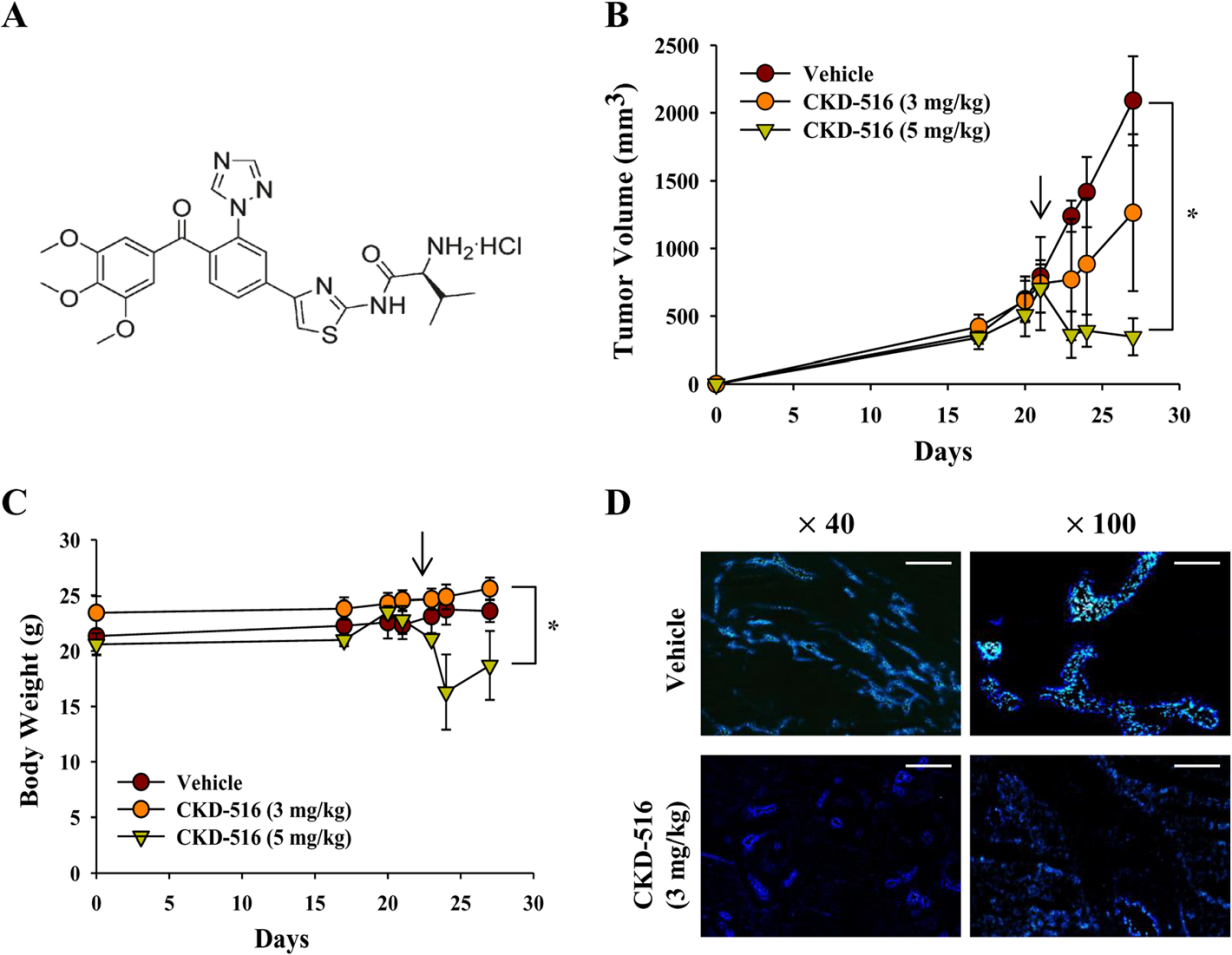
CKD-516 anti-tumor efficacy depends on the dose. **a** Molecular structure of CKD-516. **b** Quantification of tumor volume and (**c**) body weight in H520 xenograft mice following treatment with two different doses of CKD-516. Mice were divided into three groups (vehicle, PBS treated; CKD-516, 3 mg/kg; and CKD-516, 5 mg/kg) when tumor volume reached 600 mm^3^–800 mm^3^. CKD-516 was administered via intraperitoneal injection (arrow: administration of CKD-516). **d** Morphological changes in blood vessels induced by CKD-516. Heochst33342 was injected intravenously into the tail vein 72 h (at 27 days) after completion of the administration schedule. Mice were then sacrificed, and blood vessels were examined by fluorescence microscopy (*n* = 6 per group). The data are presented as the median ± IQR. Scale bar: 500 μm for × 40 and 200 μm for × 100. * denotes *p* < 0.05

We evaluated the anti-tumor efficacy of CKD-516 at 3 mg/kg and 5 mg/kg in BALB/c nude mice bearing H520 xenograft tumors. The molecular structure of CKD-516 is shown in Fig. 1a [17]. Following the completion of drug treatment, we observed that tumor volumes were reduced by 40 and 81% (*p* = 0.002) in the 3 mg/kg and 5 mg/kg CKD-516 groups, respectively, compared to the vehicle (1.3 ± 0.6 cm^3^ and 0.4 ± 0.1 cm^3^ vs. 2.1 ± 0.3 cm^3^) (Fig. 1b).

Additionally, we stained tumor tissues with Hoechst33342 to study the morphological changes caused by CKD-516 to the tumor vasculature. Using fluorescence microscopy, we observed obvious morphological changes in the blood vessel shapes of mice treated with CKD-516 (Fig. 1d). Although a high dose of CKD-516 (5 mg/kg) markedly reduced tumor volume compared to the low dose (3 mg/kg), continuous body weight loss was evident following high-dose (5 mg/kg) treatment (*p* = 0.047) (Fig. 1c). Based on these data, we chose to use 3 mg/kg CKD-516 for subsequent experiments.

### Anti-tumor efficacy of short-term CKD-516 monotherapy or combination therapy with IR

We evaluated the anti-tumor efficacy of short-term administration of CKD-516 alone or in combination with IR. To determine whether anti-tumor efficacy persisted even after treatment stopped, we compared tumor volumes 24 h and 72 h after the end of treatment.

**Fig. 2:**
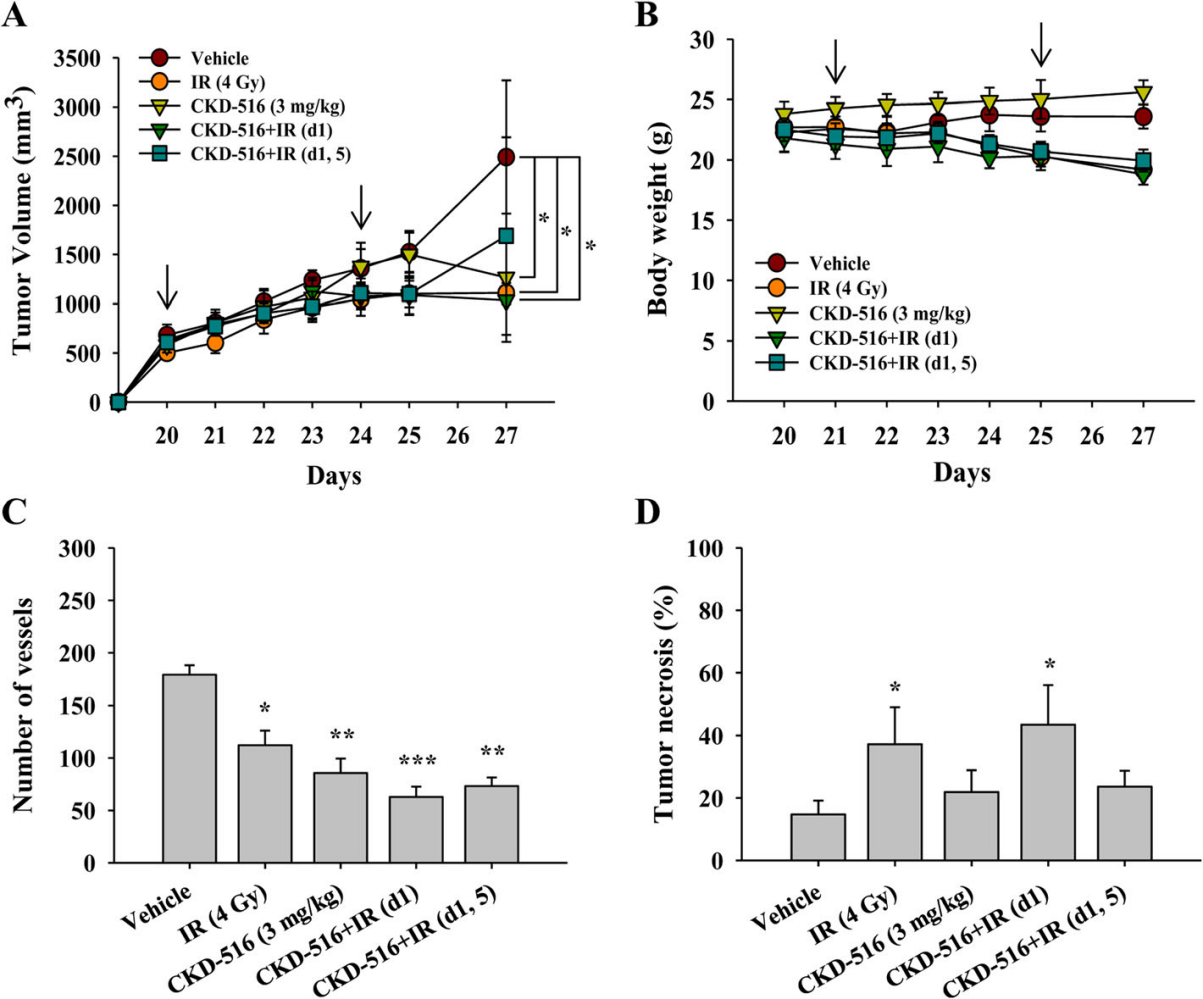
Tumor growth and vascularity are reduced, and tumor necrosis is observed following short-term combination. **a** Quantification of tumor volume and (**b**) body weight. Xenograft mice were divided into five groups according to the administration schedule: vehicle (PBS), IR alone (4 Gy/day), CKD-516 alone (3 mg/kg), and CKD-516 (3 mg/kg, day 1 or days 1 and 5) combined with IR. We irradiated the tumor mass at 4 Gy for 5 consecutive days from day 20 to day 24 when tumor volume reached 600–800 mm^3^. We investigated two treatment schedules of CKD-516 (day 1 or days 1 and 5) 1 h after IR via intraperitoneal injection (arrow: administration of CKD-516). Mice were sacrificed 24 h (25 days) and 72 h (27 days) after completion of the administration schedule (*n* = 6 per group). **c** The number of blood vessels was counted by IHC using the CD31 antibody in collected tumor tissues. **d** Histopathological analysis of tumor necrosis in mice tissues with H&E staining. The data are presented as the mean ± SE. * denotes *p* < 0.05, ** denotes *p* < 0.001, and *** denotes *p* < 0.0001

Twenty-four hours after treatment was stopped, CKD-516 alone did not induce any additional reduction in tumor volume (1.5 ± 0.7 cm^3^). However, IR treatment alone showed a further 28% reduction in tumor volume compared to the vehicle (1.1 ± 0.1 cm^3^ vs. 1.5 ± 0.7 cm^3^, *p* = 0.001; Fig. 2a). In contrast, 72 h after the end of treatment, monotherapy with IR and CKD-516 markedly decreased tumor volumes by 56% (1.1 ± 0.1 cm^3^, *p* = 0.006) and 49% (1.3 ± 0.7 cm^3^, *p* = 0.021), respectively, compared to vehicle treatment (2.5 ± 0.8 cm^3^). However, between 24 h and 72 h, IR alone did not change tumor volume; however, CKD-516 alone reduced tumor volume by 0.8-fold compared to the vehicle.

We combined IR and CKD-516 treatment using two different treatment schedules: IR (5 times per week) and CKD-516 once on day 1 (CKD-516 + IR (d1)) or twice on days 1 and 5 (CKD-516 + IR (d1, 5)) (Additional file 1). Compared to vehicle treatment, CKD-516 + IR (d1) reduced tumor volume by 29% at 24 h and by 59% at 72 h (*p* = 0.049), but there were no notable changes in tumor volume between 24 h and 72 h (1.1 ± 0.03 cm^3^ vs. 1.0 ± 0.04 cm^3^). CKD-516 + IR (d1, 5) also significantly reduced tumor volume by 28% at 24 h (1.1 ± 0.04 cm^3^, *p* = 0.024) and by 32% at 72 h (1.7 ± 1.0 cm^3^, *p* = 0.032). The tumor volumes of mice treated with CKD-516 + IR (d1) and CKD-516 + IR (d1, 5) 24 h after the end of treatment were similar to IR alone. Interestingly, after 72 h, the tumor volume was increased 1.5-fold in CKD-516 + IR (d1, 5)-treated mice. Tumor growth inhibition (TGI) and tumor growth delay (TGD) values of CKD-516 alone or CKD-516 + IR combinations were similar to those of IR alone at 24 h. However, of the two combinations, only CKD-516 + IR (d1) enhanced both TGI (59%) and TGD (31%) at 72 h (*p* = 0.049).

Notable body weight loss of 15, 14, and 12%, was observed following IR, CKD-516 + IR (d1), and CKD-516 + IR (d1, 5) treatment, respectively. In contrast, no changes in body weight were observed in the mice treated with CKD-516 alone (Fig. 2b). After counting the number of blood vessels, we found that the number of positively CD31 stained blood vessels was significantly reduced in mice treated with CKD-516 alone (52%, *p* < 0.001) compared to the vehicle (Fig. 2c). Mice treated with CKD-516 + IR (d1) and CKD-516 + IR (d1, 5) showed a 65% (*p* < 0.005) and 59% (*p* < 0.001) reduction in the number of blood vessels, respectively. We also analyzed tumor necrosis areas in tumor tissues stained with H&E and found that IR significantly increased necrosis by 60% compared to the vehicle (*p* = 0.004). Interestingly, CKD-516 + IR (d1) treatment induced the most extensive tumor necrosis (66%, *p* = 0.02) compared to the vehicle (Fig. 2d). However, tumor necrosis in the CKD-516 alone or CKD-516 + IR (d1, 5) groups did not differ significantly from the vehicle.

### Sustained tumor necrosis and hypoxia following short-term combination treatment with CKD-516 and IR

**Fig. 3:**
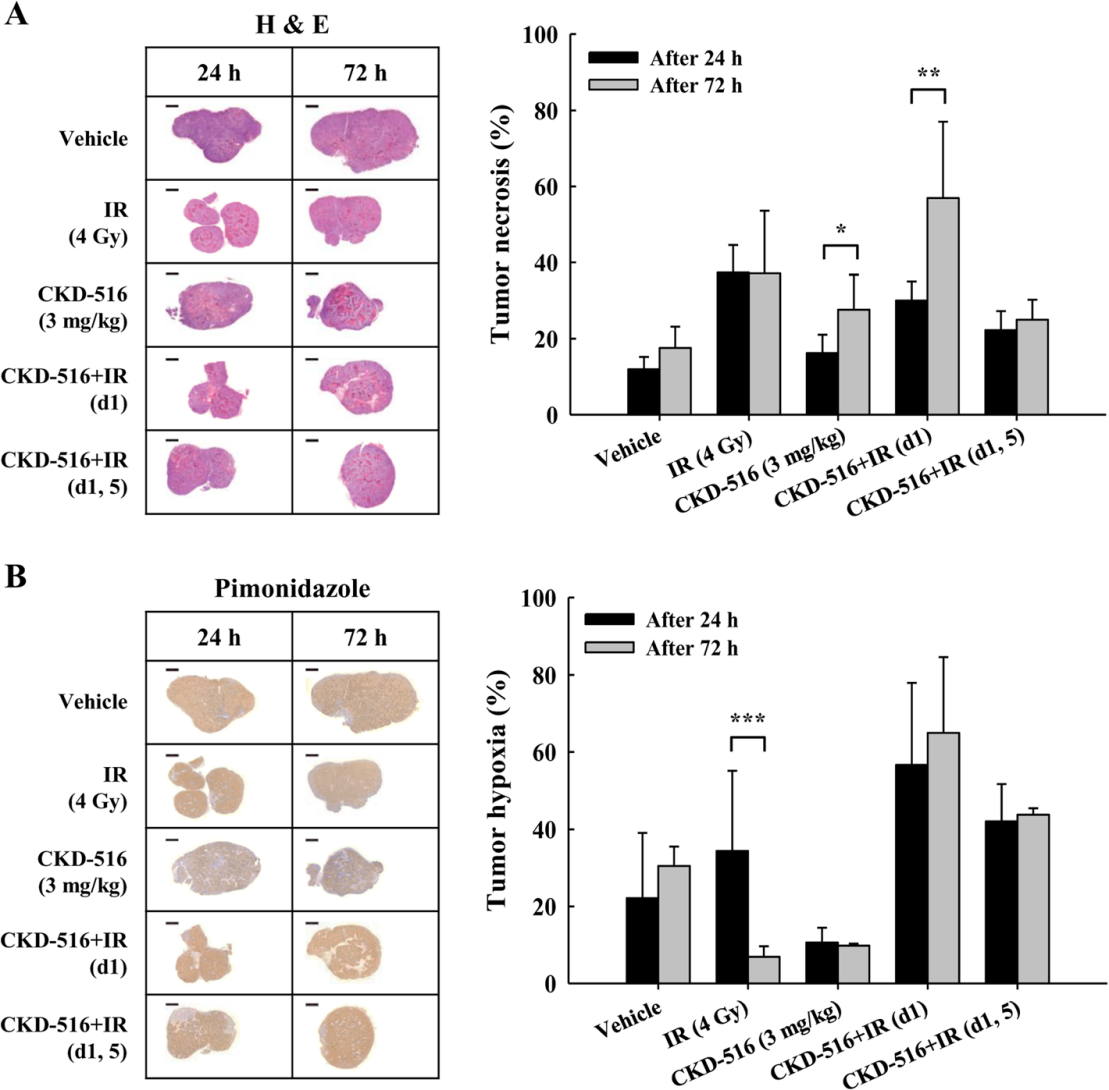
Short-term combination treatment with CKD-516 and IR induces persistent tumor necrosis and hypoxia. **a** Necrosis area by H&E staining (right panel) and quantified graph (left panel). **b** Hypoxic area by pimonidazole staining (right panel) and quantified graph (left panel). Tumor tissues obtained from sacrificed mice were stained with H&E or pimonidazole. Necrosis and areas of hypoxia were analyzed on whole slide images and the data were then compared between the five groups: vehicle (PBS), IR alone (4 Gy/day), CKD-516 alone (3 mg/kg), and CKD-516 (3 mg/kg, day 1 or days 1 and 5) combined with IR. The data are presented as the mean ± SE. * denotes *p* < 0.05, ** denotes *p* < 0.01, and *** denotes *p* < 0.001. Scale bar: 2 mm

We investigated the post-treatment effects of monotherapy and combination therapy with IR and CKD-516 on tumor necrosis and on the hypoxic tumor microenvironment. We observed the largest tumor necrosis area (%) in mice treated with IR alone (37%) 24 h after treatment. However, no further changes were detected at 72 h (37%; Fig. 3a). Treatment with both CKD-516 alone and CKD-516 + IR (d1) produced larger areas of tumor necrosis (41%, *p* = 0.049 and 47%, *p* = 0.004, respectively) after 72 h. Additionally, 24 h after the end of treatment, we measured hypoxic areas: 34% in IR alone, 57% in CKD-516 + IR (d1), and 42% in CKD-516 + IR (d1, 5) (Fig. 3b). After 72 h, the hypoxic areas rapidly decreased from 34 to 7% in mice treated with IR alone (*p* < 0.001).

### Expression of hypoxia-related proteins in mice following short-term combination treatment with CKD-516 and IR

**Fig. 4:**
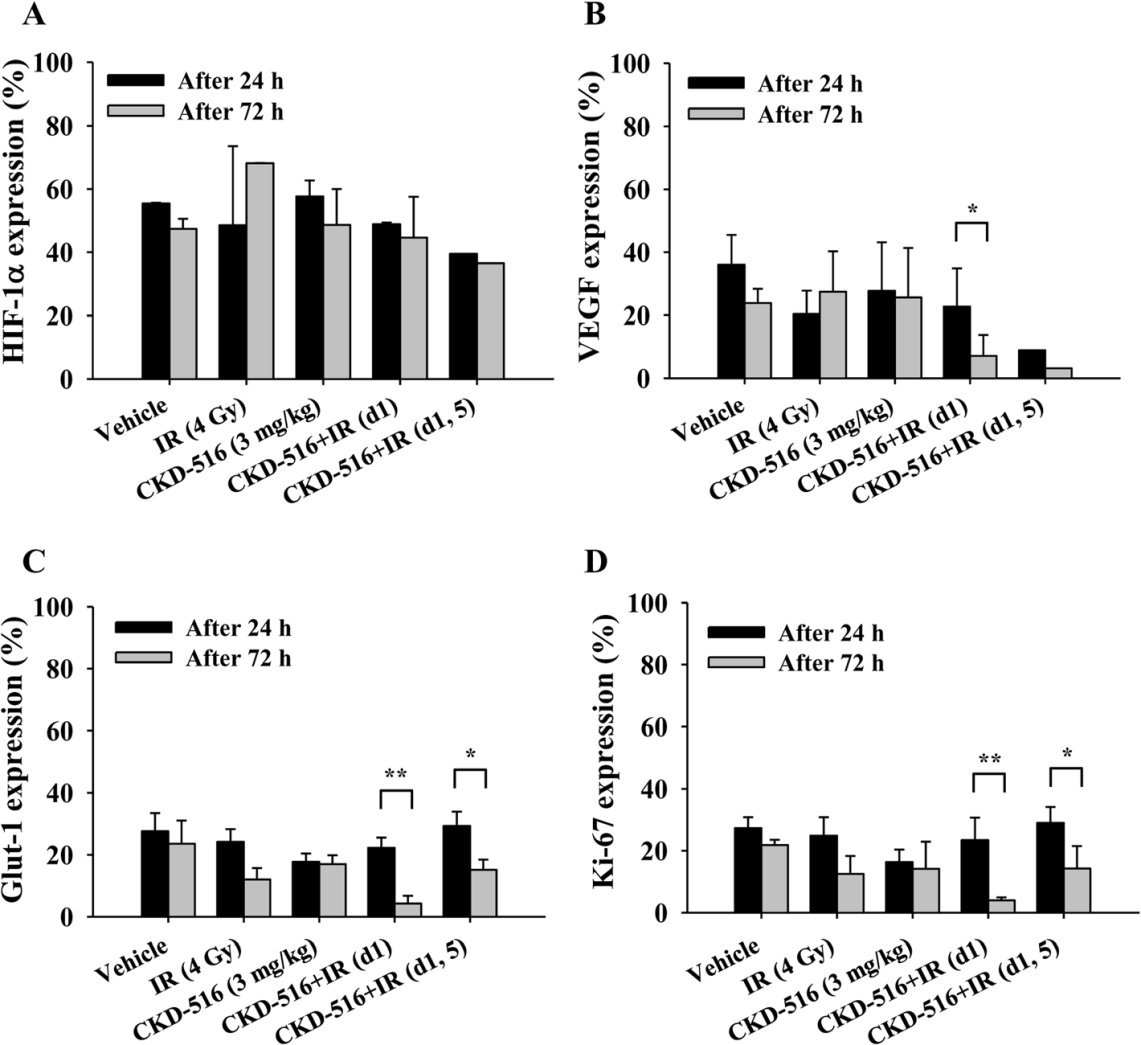
Quantitative results of IHC staining with hypoxia-related proteins following short-term combination treatment with CKD-516 and IR. **a** HIF-1α, (**b**) VEGF, (**c**) Glut-1 and (D) Ki-67. Density was analyzed according to the degree of staining. Mice were divided into five groups according to the dosing and treatment schedule: vehicle (PBS), IR alone (4 Gy/day), CKD-516 alone (3 mg/kg), and CKD-516 (3 mg/kg, day 1 or days 1 and 5) combined with IR. The data are presented as the mean ± SE. * denotes *p* < 0.05 and ** denotes *p* < 0.01

We evaluated the expression of hypoxia-related proteins (HIF-1α, Glut-1, VEGF, and Ki-67), which are involved in the maintenance of the hypoxic tumor microenvironment, in mice treated with CKD-516 and IR alone, and in combination (Additional file 3). The expression of HIF-1α, a classic marker for hypoxia, was the highest in mice treated with CKD-516 alone (58%) 24 h after treatment (Fig. 4a). However, 72 h after treatment, HIF-1α expression was highest in mice treated with IR alone (68%). VEGF expression increased by 35% 72 h after treatment with IR alone (Fig. 4b). In CKD-516 + IR (d1)-treated mice, VEGF expression decreased significantly from 22 to 7% (*p* = 0.019). Glut-1 expression decreased to 20–30% in the analyzed areas in all treatment groups 24 h after drug treatment (Fig. 4c). In mice treated with IR alone, Glut-1 expression decreased by 50% from 24 h to 72 h. Additionally, Glut-1 expression was greatly reduced in the CKD-516 + IR (d1) group (81%, *p* = 0.004). Of the four treatment groups, Ki-67 expression was the lowest (16%) in mice treated with CKD-516 alone 24 h after drug administration (Fig. 4d). However, Ki-67 expression decreased significantly in the CKD-516 + IR (d1) and CKD-516 + IR (d1, 5) groups (86%, *p* = 0.004 and 51%, *p* = 0.027, respectively) 72 h post-treatment.

### Delayed tumor growth after long-term combination treatment with CKD-516 and IR

We evaluated the effect on delayed tumor growth, tumor necrosis, and tumor hypoxia following short-term and long-term CKD-516 and IR combination treatment. Since weight loss and skin rash due to IR were frequently observed in short-term treatment (Additional file 2B and C), the IR dose was lowered from 4 Gy to 2 Gy in the long-term combination treatment schedule.

We found that 24 and 72 h after the end of treatment, IR alone decreased tumor volumes by 52% (1.5 ± 0.4 cm^3^ vs. 3.0 ± 1.1 cm^3^) and by 56% (1.6 ± 0.4 cm^3^ vs. 3.6 ± 1.3 cm^3^), respectively, compared to the vehicle. However, following treatment with CKD-516 alone, tumor volumes did not differ significantly from vehicle treatment (2.7 ± 0.9 cm^3^ vs. 3.2 ± 1.0 cm^3^, respectively).

Even though CKD-516 + IR did not cause any significant change in tumor volume between 24 h (1.2 ± 0.04 cm^3^) and 72 h (1.0 ± 0.4 cm^3^) after the end of treatment, the tumor tended to decrease, unlike other groups. Compared to IR alone, CKD-516 + IR reduced tumor volumes 1.2-fold after 24 h and 1.5-fold after 72 h (*p* < 0.001). Furthermore, when compared to CKD-516 alone, the combination treatment significantly reduced tumor volumes 2.3-fold after 24 h (vs. 2.7 ± 0.9 cm^3^, *p* = 0.001) and 3.1-fold after 72 h (vs. 3.2 ± 1.0 cm^3^, *p* < 0.001).

We found that compared to mice treated with IR or CKD-516 alone, the TGI and TGD values (%) 72 h after CKD-516 + IR treatment were 1.3- and 1.9-fold higher than those of IR alone (71% vs. 56%, p = 0.001 and 28% vs. 15%, *p* = 0.004, respectively). Compared to mice treated with CKD-516 alone, the TGI values of CKD-516 + IR at 24 h and 72 h were 6.3- and 6.8-fold higher (*p* = 0.002), and TGD values were 14.5- and 20.6-fold higher (p = 0.001), respectively (Table 1).

**Table 1:** Tumor growth inhibition and tumor growth delay by CKD-516 combined with IR

Groups	Radiation	CKD-516	Short-term (1 cycle) (***n*** = 6)				Long-term (3 cycles) (***n*** = 10)			
			% TGI		% TGD (2000 mm^**3**^)		% TGI		% TGD (2000 mm^**3**^)	
			24 h	72 h	24 h	72 h	24 h	72 h	24 h	72 h
**Vehicle**	**–**	**–**	–	–	–	–	–	–	–	–
**IR**	**2 Gy**	**–**	–	–	–	–	51.9^**^	56.2^**^	15.1^**^	14.9^**^
	**4 Gy**	**–**	27.8^**^	55.5^*^	12.6^**^	27.0^*^	–	–	–	–
**CKD-516**	**–**	**3 mg/kg**	1.5	49.2	0.5	21.0	9.6	10.5	1.5	1.4
**CKD-516** **+ IR (d1)**	**2 Gy**	**3 mg/kg**	–	–	–	–	60.9^**^	71.2^**^	21.7^**^	28.8^**^
	**4 Gy**	**3 mg/kg**	28.6	58.5^*^	13.2	30.6^*^				
CKD-516 **+ IR (d1, 5)**	**4 Gy**	**3 mg/kg**	27.9^*^	32.2^*^	12.7^*^	10.3^*^				

**Notes**: *IR* Radiation, *TGI* Tumor growth inhibition, *TGD* Tumor growth delay. *denotes *p* < 0.05, **denotes *p* < 0.01. % TGI = 100 - (T/C × 100), where T = mean tumor volume of treatment group and C = mean tumor volume of vehicle group. % TGD = (T - C)/C × 100, where T = median time to endpoint of treatment group and C = median time to endpoint of vehicle group

**Fig. 5:**
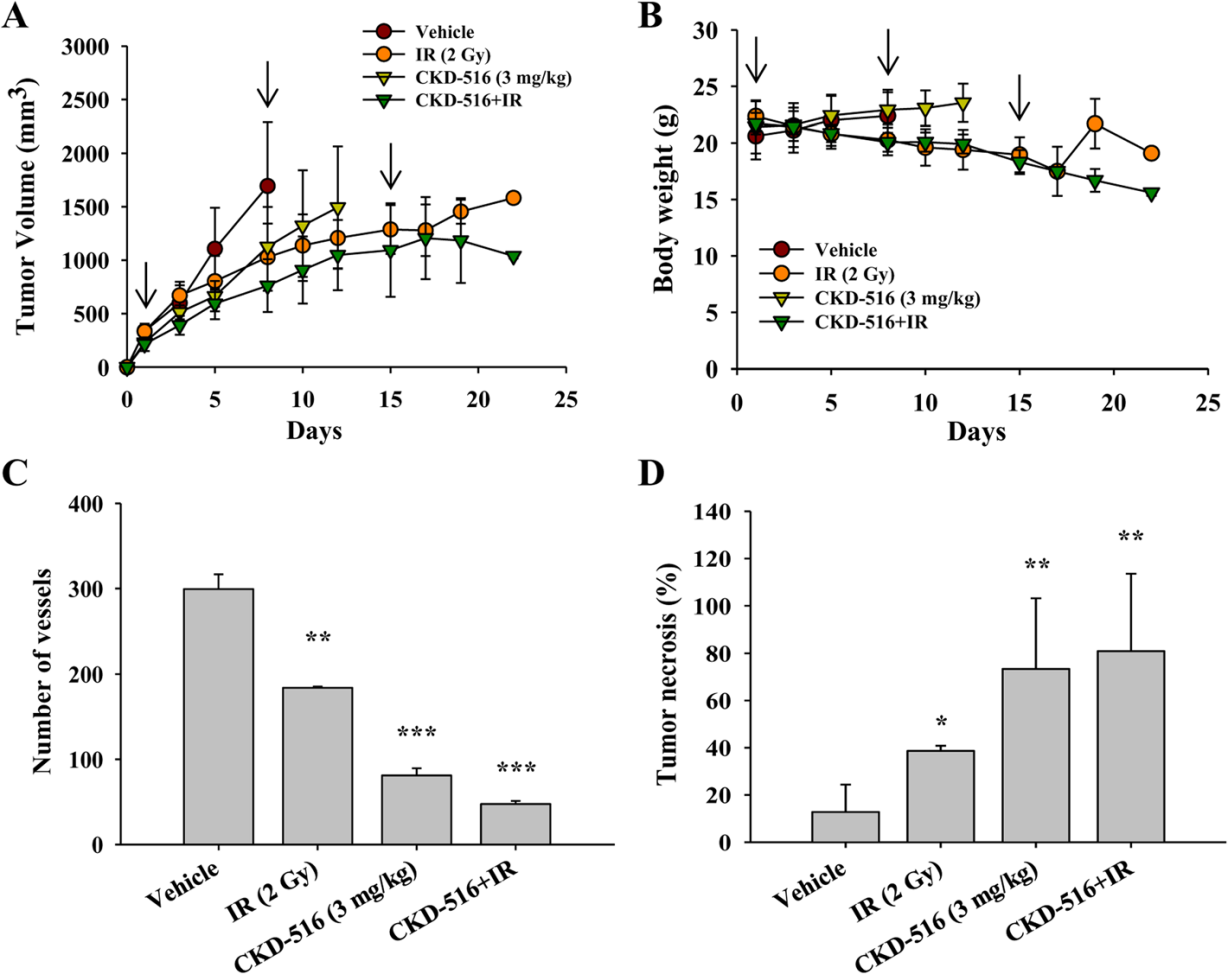
Tumor growth is suppressed and delayed following long-term combination treatment with CKD-516 and IR. **a** Quantification of tumor volume and (**b**) body weight after long-term treatment monotherapy with IR and CKD-516. H520 cells were injected subcutaneously into the right forearm of nude mice. Depending on the administration method, mice were divided into four groups: vehicle (PBS), IR alone (2 Gy/day), CKD-516 (3 mg/kg) alone, and CKD-516 (3 mg/kg, day 1) combined with IR. IR was administered at 2 Gy every 3 weeks for 5 days per cycle and CKD-516 was administered via intraperitoneal injection 1 h after IR on the first day of the cycle (arrow: CKD-516 administration). At the end of each cycle, no treatment was administered for 2 days. Mice were sacrificed 72 h (22 days) after the end of the administration schedule. **c** CD31 was used to stain tumor tissues using IHC. The number of blood vessels was measured for each group. **d** The area of tumor necrosis was analyzed by H&E staining (*n* = 10 per group). The data are presented as the mean ± SE. * denotes *p* < 0.05, ** denotes *p* < 0.01, and *** denotes *p* < 0.001

No significant differences in body weight were found between mice treated with CKD-516 alone and the vehicle group (Fig. 5b). However, both IR alone and CKD-516 + IR groups showed a gradual decrease in body weight as the administration schedule progressed. After measuring the number of blood vessels 72 h after the end of treatment, we found that compared to the vehicle, CKD-516 alone, IR alone, and CKD-516 + IR groups had a significantly reduced number of blood vessels (38%, *p* = 0.003; 73%, *p* < 0.001; and 84%, *p* < 0.001, respectively; Fig. 5c). Conversely, the tumor necrosis area increased significantly to 67% in the IR alone group, 82% in the CKD-516 alone group, and 84% in the CKD-516 + IR group compared to the vehicle group (*p* = 0.02, *p* = 0.005, and *p* = 0.004, respectively; Fig. 5d).

### Hypoxia-related protein expression in mice following long-term combination treatment with CKD-516 and IR

**Fig. 6:**
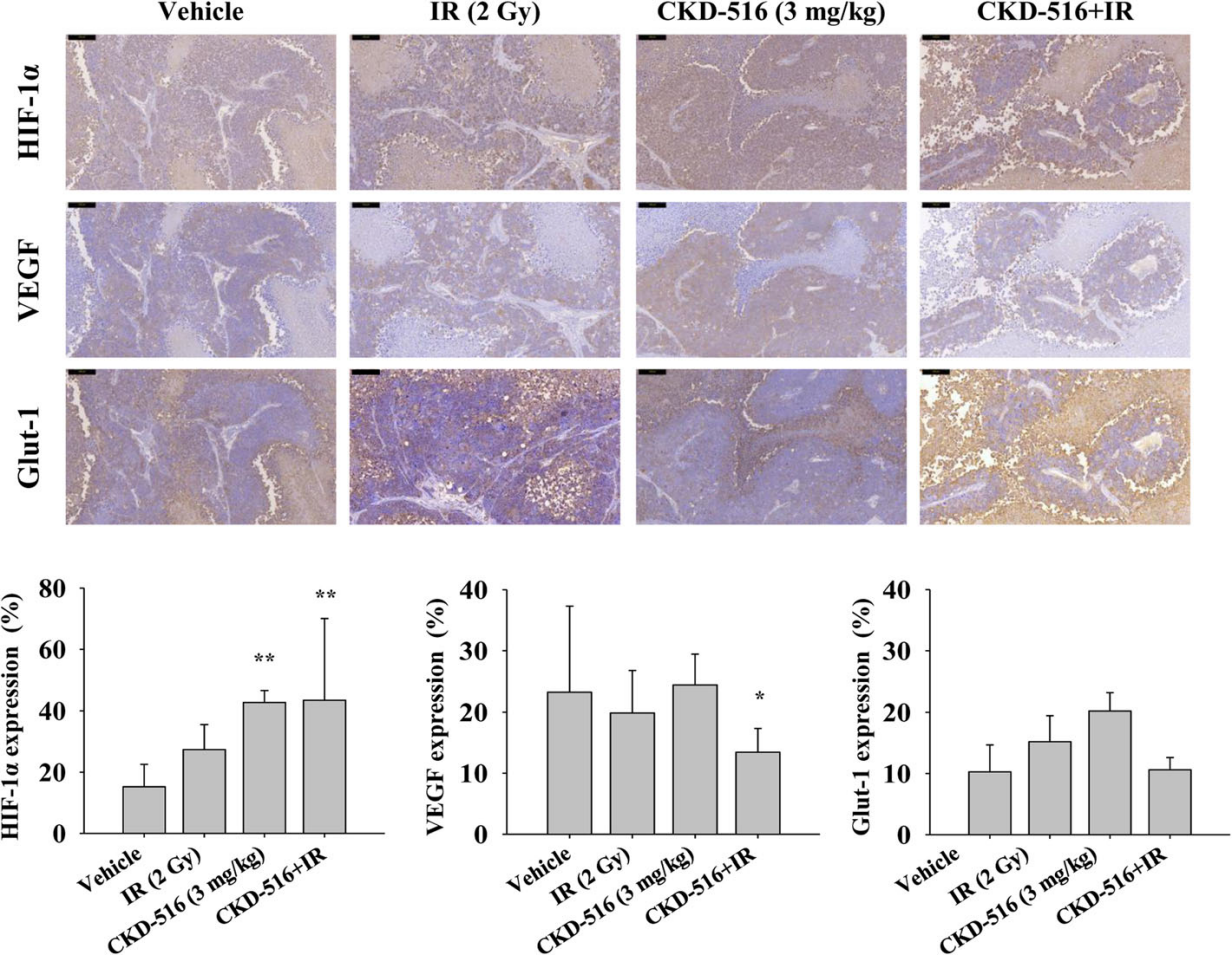
Changes in the expression of hypoxia-related proteins after three cycles of CKD-516 combined with IR. Qualitative (top) and quantitative (bottom) expression of hypoxia-related proteins, including HIF-1α, VEGF, and Glut-1 after IHC staining. Mice were divided into four groups according to the administration methods: vehicle (PBS), IR alone (2 Gy/day), CKD-516 alone (3 mg/kg), and CKD-516 (3 mg/kg, day 1) combined with IR (n = 10 per group). The data are presented as the mean ± SE. * denotes *p* < 0.05 and ** denotes *p* < 0.01. Scale bar: 100 μm. Magnification: × 20

HIF-1α expression increased significantly by 64% in the CKD-516 alone group (*p* = 0.002) and by 65% in the CKD-516 + IR group (*p* = 0.005) compared to the vehicle (Fig. 6a). VEGF expression in both IR alone and CKD-516 alone groups was similar to that in the vehicle group. However, VEGF expression was significantly lower (41%, *p* = 0.046) in the CKD-516 + IR group (Fig. 6b). Glut-1 expression was upregulated in mice treated with IR alone and in combination with CKD-516 alone. However, there were no significant changes in the CKD-516 + IR group (Fig. 6c). Ki-67 expression was considerably diminished in the IR alone, CKD-516 alone, and CKD-516 + IR groups (4, 4, and 5%, respectively) (data not shown).

## Discussion

Combination treatment with chemotherapy and IR has widely been accepted as the standard treatment for locally advanced stage III NSCLC. However, hypoxic and acidic areas in the center of tumors can lead to radiation resistance, a major cause of treatment failure. To overcome the IR-induced tolerance of hypoxic conditions, many studies have combined VDA or angiogenesis inhibitor treatment with IR [19–23]. Although VDAs can cause rapid occlusion in the central tumor vessel, VDA drug resistance can occur immediately [24]. This could be caused by the remaining cancer cells adapting to acquire nutrients and oxygen from the marginal areas of the tumor [25]. Since tumor growth is restored within a few hours of VDA treatment [26, 27], it is very important to combine VDAs with other treatments to improve their antitumor efficacy. Preclinical studies have shown that CKD-516 is an excellent tool for disrupting tumor vasculature [28–30]. CKD-516 has also been shown to be safe in early clinical studies [31]. Recently, several investigators have demonstrated synergistic antitumor efficacy by combining CKD-516 with other cytotoxic agents such as doxorubicin or gemcitabine in hepatocellular carcinoma and lung cancer xenograft mice [32, 33]. In a preliminary study of tumorigenesis in SK-MES-1, HCC-95, and H520 SqCC cell lines in nude mice, H520 cells showed the greatest potential for tumor formation. We evaluated antitumor efficacy by measuring changes in the expression of hypoxia-related signaling molecules in SqCC xenograft mice after short- and long-term administration of CKD-516 alone or in combination with IR.

The results of the present study confirm that 5 mg/kg CKD-516 reduces tumor volume and increases tumor necrosis significantly more than at the lower dose of 3 mg/kg. There were no noticeable changes in body weight after low-dose treatment; however, gradual weight loss was observed after high-dose treatment. Therefore, we used 3 mg/kg CKD-516 for subsequent experiments. Preclinical data have previously shown that VDA administration following IR is more effective at inhibiting tumor growth in a breast cancer model [19]. Therefore, in the present study, CKD-516 was also administered 1 h after IR.

After short-term 1-week treatment with CKD-516 alone, IR alone, or their combination, we found that both IR alone and CKD-516 + IR (d1) significantly reduced tumor volumes by more than 50%. More specifically, CKD-516 + IR (d1) inhibited tumor growth up to 72 h after treatment; however, this was not the case in CKD-516 + IR (d1, 5) where the tumor continued to grow. There was less tumor necrosis and hypoxia with increased expression of Glut-1 and Ki-67 in the CKD-516 + IR (d1, 5) group compared to the CKD-516 + IR (d1) group. Interestingly, we found that the expression of Ki-67 in the rim area of tumor tissue in CKD-516 + IR (d1, 5) increased (data not shown). Therefore, tumors were more likely to start proliferating again in CKD-516 + IR (d1, 5) 72 h after the end of drug treatment.

In our study, the combination treatment of CKD-516 + IR significantly reduced the number of vessels and CKD-516 + IR (d1) produced the most extensive tumor necrosis. In a previous study using a hepatocellular carcinoma xenograft model, CKD-516 caused necrosis in the central area of the tumor and markedly reduced CD31 expression [33]. These previous studies agree with our data. We investigated the delayed effects on tumor necrosis and hypoxia caused by CKD-516 alone, IR alone, and their combinations 72 h after treatment. Mice treated with CKD-516 alone and CKD-516 + IR (d1) showed significantly enhanced tumor necrosis between 24 h and 72 h. CKD-516 + IR (d1) induced the largest hypoxic area among all treatment groups at 24 h. Moreover, these hypoxic areas grew even larger 72 h after treatment. Previous studies have reported that the VDA, combretastatin A-4-P (CA-4-P), can cause tumor necrosis up to 120 h after treatment cessation and decrease hypoxia at 24 h [34]. In NSCLC xenograft mice, CA-4-P induced vascular shutdown and necrosis between 1 h and 3 h after drug treatment [35]. In our study, tumor hypoxia and necrosis continued even 24–72 h after the end of treatment. This finding suggests that CKD-516 induces hypoxia and necrosis over a longer period of time. After checking the expression of hypoxia-related proteins, we found that in the short-term treatment schedule, CKD-516 + IR and, more specifically, CKD-516 + IR (d1) treatment, decreased HIF-1α, Glut-1, and VEGF expression continuously from 24 h to 72 h. Additionally, CKD-516 + IR (d1) markedly reduced Ki-67 expression up to 72 h. Our data suggest that combination drug treatment with CKD-516 (d1) and IR (d1–5) is the most effective treatment schedule for reducing tumor volume and inducing tumor necrosis. In fact, CKD-516 (d1) alone induces central tumor necrosis by vascular occlusion with decreased VEGF, Glut-1, and Ki-67 expression. Additional administration of CKD-516 (d5) did not seem to have a synergistic effect with IR.

Our results contradict prior studies showing that the expression of hypoxia-related proteins such as HIF-1α, VEGF, and Glut-1 is upregulated under hypoxic conditions [8, 9, 36, 37]. In contrast, another study has shown that potent VEGF inhibitors, including sunitinib and ziv-aflibercept, cause tumor necrosis and downregulate CD31 and Ki-67 expression in the renal cell carcinoma PDX model, which was consistent with our results [38]. The most likely explanation for our result is that CKD-516 + IR rapidly reduces tumor blood flow under excessive hypoxic conditions, leading to rapid tumor cell death with decreased VEGF, Glut-1, and Ki-67 expression.

In the present study, loss of body weight and skin rashes were recorded in all IR-treated groups, with no significant differences between the groups. We constructed lead shields for local radiation of the tumor-bearing site only. However, in the case of Cs-137, the minimum lead thickness required for complete shielding from radiation is 3.5 cm. However, because the radiation equipment used in this study could not contain the aforementioned shield, the experiment was conducted with a lead shield of 4 mm, the maximum thickness possible. The energy of Cs-137 is reduced by approximately 50% in the 7 mm thick lead shield [39]. Hence, the energy reduction by the 4 mm thick lead shield is estimated to be approximately 30%. Upon long-term treatment with 4 Gy of IR, we observed serious body weight loss in mice (Additional file 2D). We hypothesize that this may have been because the lead shield did not completely prevent whole-body IR of the mice. Prior in vivo studies have shown that if the body weight of C57BL/6 mice falls by approximately 13–20%, they are less likely to survive [40]. Furthermore, BALB/c nude mice responded more sensitively to IR than C57BL/6 mice [41]. One study has shown that when IR is administered to colon cancer xenograft mice at 2 Gy for 5 days, tumor volume is reduced while body weight remains unchanged [42]. Practically speaking, the dose schedule of our experiment was conventional (2 Gy/fx) and hypofractionated RT (4 Gy/fx). Therefore, we investigated a total of 30 Gy of radiation with 2 Gy/15 fractions of conventionally fractionated radiation therapy (CFRT) in long-term treatment. This is a low dose when the subject is to complete local control, but it is not a small dose for evaluating the combined anticancer efficacy of CKD-516 + IR in mice. CFRT remains the most common radiotherapy used in many patients with solid tumors; however, recent studies have reported that stereotactic body radiation therapy (SBRT) has been demonstrating improved therapeutic effects and overall survival when compared to CFRT [43]. Thus, further studies on anticancer efficacy combined with novel IR techniques are needed. In this experiment, it took about 5 min after anesthesia to complete the irradiation. Even though we were unable to accurately quantify the drop in body temperature of the mice during irradiation, there were no remarkable differences in temperatures before and after irradiation. However, thermal regulation in anesthetized mice is generally poor and hypothermia is likely to change blood pressure and blood flow, eventually leading to increased hypoxia in the tumor bed. Hence, hypothermia needs to be prevented by administering fluid subcutaneously or peritoneal in order to facilitate blood circulation even under anesthesia, or by supplying heat through a heat pad or infrared lamp during irradiation [44, 45].

Long-term treatment with single IR and CKD-516 + IR significantly inhibited tumor growth, resulting in markedly reduced tumor volumes. Moreover, CKD-516 + IR delayed tumor growth up to 72 h after cessation of treatment. A previous report showed that tumor growth was delayed for up to 240 days following IR monotherapy in a lung cancer xenograft rat model [46]. Another study reported that the combination of CKD-516 and gemcitabine can greatly enhance its anti-cancer efficacy [32]. Additionally, we found that tumor growth was restored 3 days after a single administration of CKD-516. However, tumor growth has also been shown to be effectively inhibited for up to 7 days after combination therapy with CKD-516 and doxorubicin [33]. These results strongly support our conclusion that the CKD-516 + IR combination treatment could significantly reduce tumor volumes by delaying the tumor growth rate. However, considering the continuous decrease in body weight during long-term treatment, the IR dose should be readjusted in follow-up studies.

In the current study, treatment with IR alone and CKD-516 + IR significantly decreased the number of blood vessels, while CKD-516 + IR increased central tumor necrosis with a larger range, similar to results obtained from the short-term treatment schedule. Both CKD-516 alone or CKD-516 + IR significantly increased HIF-1α expression. However, CKD-516 + IR noticeably decreased VEGF and Glut-1 expression. Ki-67 expression was also significantly reduced when CKD-516 was combined with IR (data not shown). Glut-1, a glucose transporter regulated by HIF-1α, is involved in the inhibition of cell death together with other glucose-degrading proteins [47]. Previous studies have shown that Glut-1 and VEGF expression are upregulated when HIF-1α expression increases [36, 37]. When Glut-1 expression decreases, Ki-67 expression is downregulated [48]. However, in our study, Ki-67 expression increased in the peripheral margins compared to the central portion of the tumor (data not shown). Several studies have shown that Ki-67 expression typically decreases 24 h after treatment with OXi4503, a tubulin binding agent, but increases again in the tumor margins at day 5 post-treatment [27]. Resistance to VDAs due to the rebound of proliferating cancer cells at the tumor margins is a major obstacle in cancer treatment strategies. Further studies are urgently needed to better understand the underlying mechanisms of this rebounding effect. Overall, decreased Ki-67 expression, as well as prolonged inhibition of VEGF and Glut-1 expression, by long-term administration of CKD-516 + IR, may be due to a sustained hypoxic microenvironment and central tumor necrosis. In a subcutaneous model of in vivo lung cancer, vascular permeability and perfusion are lower than those in the orthotopic model [49]. Another study reported that median oxygenation in tumor tissues of NSCLC patients was higher when compared to that in other solid tumors [50]. Accordingly, to increase accuracy in the assessment of antitumor efficacy and to generate results of radiation therapy associated with hypoxia, it is important to establish a tumor model with conditions similar to those of human lung cancer. Cancer research using an orthotopic mouse model constructed by surgical intervention [51, 52] will be useful in overcoming some of the aforementioned limitations associated with in vivo tumor models.

## Conclusion

Taken together, our results suggest that combination therapy with CKD-516 and IR delays tumor growth with extensive central necrosis compared to CKD-516 or IR monotherapy in an in vivo SqCC xenograft model. Further studies are required to overcome some limitations of VDAs.

## Supplementary Information

**Additional file 1.** Short-term and long-term drug administration schedules.

**Additional file 2.** Toxicity of low- or high-dose IR in BALB/c nude mice. (A) The shielding device was made of 4 mm thick lead. The anesthetized mice were fixed in a 50 mL tube and irradiated with a lead shield. (B) Tumor growth and (C) body weight according to radiation dose. (D) Tumor growth during long-term treatment with 4 Gy of IR.

**Additional file 3.** IHC staining with HIF-1α, VEGF, Glut-1, and Ki-67 antibodies in all five treatment groups (72 h after the end of treatment). Mice were divided into five groups according to the dosing and treatment schedule: vehicle (PBS), IR alone (4 Gy/day), CKD-516 alone (3 mg/kg), and CKD-516 (3 mg/kg, day 1 or days 1 and 5) combined with IR.Scale bar: 200 μm. Magnification: × 100.

## Data Availability

The datasets used and/or analyzed during the current study are available from the corresponding author upon reasonable request.

## Abbreviations

CD31: Cluster of differentiation 31 (platelet/endothelial cell adhesion molecule-1)
d1, 5: Day 1 and day 5
d1: Day 1
Glut-1: Glucose transporter type 1
HIF-1α: Hypoxia-inducible factor-1 alpha
IR: Irradiation
NSCLC: Non-small cell lung cancer
PDX: Patient-derived xenograft
SCLC: Small cell lung cancer
SqCC: Squamous cell carcinoma
VDA: Vascular disrupting agent
VEGF: Vascular endothelial growth factor

## References

[1] Bonomi PD (2010). Implications of key trials in advanced non-small cell lung cancer. Cancer..

[2] Molina JR, Yang P, Cassivi SD, Schild SE, Adjei AA (2008). Non-small cell lung cancer: epidemiology, risk factors, treatment, and survivorship. Mayo Clin Proc.

[3] Oser MG, Niederst MJ, Sequist LV, Engelman JA (2015). Transformation from non-small-cell lung cancer to small-cell lung cancer: molecular drivers and cells of origin. Lancet Oncol.

[4] Mok TSK, Wu YL, Kudaba I, Kowalski DM, Cho BC, Turna HZ (2019). Pembrolizumab versus chemotherapy for previously untreated, PD-L1-expressing, locally advanced or metastatic non-small-cell lung cancer (KEYNOTE-042): a randomised, open-label, controlled, phase 3 trial. Lancet..

[5] Koh PK, Faivre-Finn C, Blackhall FH, De Ruysscher D (2012). Targeted agents in non-small cell lung cancer (NSCLC): clinical developments and rationale for the combination with thoracic radiotherapy. Cancer Treat Rev.

[6] Gray JE, Villegas A, Daniel D, Vicente D, Murakami S, Hui R (2020). Three-year overall survival with Durvalumab after chemoradiotherapy in stage III NSCLC—update from PACIFIC. J Thorac Oncol.

[7] Le QT, Chen E, Salim A, Cao H, Kong CS, Whyte R (2006). An evaluation of tumor oxygenation and gene expression in patients with early stage non-small cell lung cancers. Clin Cancer Res.

[8] Liu Y, Cox SR, Morita T, Kourembanas S (1995). Hypoxia regulates vascular endothelial growth factor gene expression in endothelial cells. Circ Res.

[9] Forsythe JA, Jiang BH, Iyer NV, Agani F, Leung SW, Koos RD (1996). Activation of vascular endothelial growth factor gene transcription by hypoxia-inducible factor 1. Mol Cell Biol.

[10] Hammond EM, Asselin MC, Forster D, O’Connor JP, Senra JM, Williams KJ (2014). The meaning, measurement and modification of hypoxia in the laboratory and the clinic. Clin Oncol (R Coll Radiol).

[11] Baguley BC (2011). Preclinical efficacy of vascular disrupting agents in non-small-cell lung cancer. Clin Lung Cancer.

[12] Tozer GM, Prise VE, Wilson J, Cemazar M, Shan S, Dewhirst MW (2001). Mechanisms associated with tumor vascular shut-down induced by combretastatin A-4 phosphate: intravital microscopy and measurement of vascular permeability. Cancer Res.

[13] El-Emir E, Boxer GM, Petrie IA, Boden RW, Dearling JL, Begent RH (2005). Tumor parameters affected by combretastatin A-4 phosphate therapy in a human colorectal xenograft model in nude mice. Eur J Cancer.

[14] Sheng Y, Hua J, Pinney KG, Garner CM, Kane RR, Prezioso JA (2004). Combretastatin family member OXI4503 induces tumor vascular collapse through the induction of endothelial apoptosis. Int J Cancer.

[15] Boehle AS, Sipos B, Kliche U, Kalthoff H, Dohrmann P (2001). Combretastatin A-4 prodrug inhibits growth of human non-small cell lung cancer in a murine xenograft model. Ann Thorac Surg.

[16] Grisham RN, Ky B, Tewari KS, Chaplin DJ, Walker J, Grisham R (2018). Clinical trial experience with CA4P anticancer therapy: focus on efficacy, cardiovascular adverse events, and hypertension management. Gynecol Oncol Res Pract.

[17] Lee J, Kim SJ, Choi H, Kim YH, Lim IT, Yang HM (2010). Identification of CKD-516: a potent tubulin polymerization inhibitor with marked antitumor activity against murine and human solid tumors. J Med Chem.

[18] Szadvari I, Krizanova O, Babula P (2016). Athymic nude mice as an experimental model for cancer treatment. Physiol Res.

[19] Iversen AB, Busk M, Horsman MR (2013). Induction of hypoxia by vascular disrupting agents and the significance for their combination with radiation therapy. Acta Oncol.

[20] Ning S, Laird D, Cherrington JM, Knox SJ (2002). The antiangiogenic agents SU5416 and SU6668 increase the antitumor effects of fractionated irradiation. Radiat Res.

[21] Clémenson C, Jouannot E, Merino-Trigo A, Rubin-Carrez C, Deutsch E (2013). The vascular disrupting agent ombrabulin (AVE8062) enhances the efficacy of standard therapies in head and neck squamous cell carcinoma xenograft models. Investig New Drugs.

[22] Ahmed B, Landuyt W, Griffioen AW, Van Oosterom A, Van den Bogaert W, Lambin P (2006). In vivo antitumor effect of combretastatin A-4 phosphate added to fractionated irradiation. Anticancer Res.

[23] Raben D, Bianco C, Damiano V, Bianco R, Melisi D, Mignogna C (2004). Antitumor activity of ZD6126, a novel vascular-targeting agent, is enhanced when combined with ZD1839, an epidermal growth factor receptor tyrosine kinase inhibitor, and potentiates the effects of radiation in a human non-small cell lung cancer xenograft model. Mol Cancer Ther.

[24] Wu XY, Ma W, Gurung K, Guo CH (2013). Mechanisms of tumor resistance to small-molecule vascular disrupting agents: treatment and rationale of combination therapy. J Formos Med Assoc.

[25] Siemann DW, Chaplin DJ, Horsman MR (2004). Vascular-targeting therapies for treatment of malignant disease. Cancer..

[26] Salmon BA, Siemann DW (2007). Characterizing the tumor response to CA4P treatment. Int J Radiat Oncol Biol Phys.

[27] Nguyen L, Fifis T, Malcontenti-Wilson C, Chan LS, Costa PN, Nikfarjam M (2012). Spatial morphological and molecular differences within solid tumors may contribute to the failure of vascular disruptive agent treatments. BMC Cancer.

[28] Il J, Lee JM, Han JK, Choi BI (2014). Intravoxel incoherent motion diffusion-weighted MR imaging for monitoring the therapeutic efficacy of the vascular disrupting agent CKD-516 in rabbit VX2 liver tumors. Radiology..

[29] Joo I, Lee JM, Grimm R, Han JK, Choi BI (2016). Monitoring vascular disrupting therapy in a rabbit liver tumor model: relationship between tumor perfusion parameters at IVIM diffusion-weighted MR imaging and those at dynamic contrast-enhanced MR imaging. Radiology..

[30] Kim KW, Lee JM, Jeon YS, Lee IJ, Choi Y, Park J (2013). Vascular disrupting effect of CKD-516: preclinical study using DCE-MRI. Investig New Drugs.

[31] Oh DY, Kim TM, Han SW, Shin DY, Lee YG, Lee KW (2016). Phase I study of CKD-516, a novel vascular disrupting agent, in patients with advanced solid tumors. Cancer Res Treat.

[32] Moon CH, Lee SJ, Lee HY, Dungle TK, Cho WJ, Cha H (2014). CKD-516 displays vascular disrupting properties and enhances anti-tumor activity in combination with chemotherapy in a murine tumor model. Investig New Drugs.

[33] Kim YI, Kim KW, Lee HK, Park J, Chung JW, Youn H (2014). Enhanced efficacy of CKD-516 in combination with doxorubicin: pre-clinical evaluation using a hepatocellular carcinoma xenograft model. Anticancer Res.

[34] Taylor M, Billiot F, Marty V, Rouffiac V, Cohen P, Tournay E (2012). Reversing resistance to vascular-disrupting agents by blocking late mobilization of circulating endothelial progenitor cells. Cancer Discov.

[35] Dey S, Kumari S, Kalainayakan SP, Campbell J, Ghosh P, Zhou H (2017). The vascular disrupting agent combretastatin A-4 phosphate causes prolonged elevation of proteins involved in heme flux and function in resistant tumor cells. Oncotarget.

[36] Inglis DJ, Lavranos TC, Beaumont DM, Leske AF, Brown CK, Hall AJ (2014). The vascular disrupting agent BNC105 potentiates the efficacy of VEGF and mTOR inhibitors in renal and breast cancer. Cancer Biol Ther.

[37] Lin Z, Weinberg JM, Malhotra R, Merritt SE, Holzman LB, Brosius FC (2000). GLUT-1 reduces hypoxia-induced apoptosis and JNK pathway activation. Am J Physiol Endocrinol Metab.

[38] Miles KM, Seshadri M, Ciamporcero E, Adelaiye R, Gillard B, Sotomayor P (2014). DII4 blockade potentiates the anti-tumor effects of VEGF inhibition in renal cell carcinoma patient-derived xenografts. PLoS One.

[39] Bakshi J (2018). Clearview radiation shielding. Radium incorporated.

[40] Nunamaker EA, Artwohl JE, Anderson RJ, Fortman JD (2013). Endpoint refinement for total body irradiation of C57BL/6 mice. Comp Med.

[41] Hanson WR, Fry RJ, Sallese AR, Frischer H, Ahmad T, Ainsworth EJ (1987). Comparison of intestine and bone marrow radiosensitivity of the BALB/c and the C57BL/6 mouse strains and their B6CF1 offspring. Radiat Res.

[42] Kim JS, Son Y, Bae MJ, Lee M, Lee CG, Jo WS (2015). Administration of granulocyte colony-stimulating factor with radiotherapy promotes tumor growth by stimulating vascularization in tumor-bearing mice. Oncol Rep.

[43] Videtic GM, Hu C, Singh AK, Chang JY, Parker W, Olivier KR (2015). A randomized phase 2 study comparing 2 stereotactic body radiation therapy schedules for medically inoperable patients with stage i peripheral non-small cell lung cancer: NRG oncology RTOG 0915 (NCCTG N0927). Int J Radiat Oncol Biol Phys.

[44] Mueller Klieser W, Vaupel P, Kallman RF (1987). Spontaneous variations of blood supply and tissue temperature in peripheral subcutaneous tumors during anesthesia. Rodent tumor models in experimental cancer therapy.

[45] Gargiulo S, Greco A, Gramanzini M, Esposito S, Affuso A, Brunetti A (2012). Mice anesthesia, analgesia, and care, part I: anesthetic considerations in preclinical research. ILAR J.

[46] Zhou H, Zhang Z, Denney R, Williams JS, Gerberich J, Stojadinovic S (2017). Tumor physiological changes during hypofractionated stereotactic body radiation therapy assessed using multi-parametric magnetic resonance imaging. Oncotarget.

[47] Malhotra R, Brosius FC (1999). Glucose uptake and glycolysis reduce hypoxia-induced apoptosis in cultured neonatal rat cardiac myocytes. J Biol Chem.

[48] Maki Y, Soh J, Ichimura K, Shien K, Furukawa M, Muraoka T (2013). Impact of GLUT1 and Ki-67 expression on early-stage lung adenocarcinoma diagnosed according to a new international multidisciplinary classification. Oncol Rep.

[49] Graves EE, Vilalta M, Cecic IK, Erler JT, Tran PT, Felsher D (2010). Hypoxia in models of lung cancer: implications for targeted therapeutics. Clin Cancer Res.

[50] Vilalta M, Hughes NP, Von Eyben R, Giaccia AJ, Graves EE (2017). Patterns of vasculature in mouse models of lung cancer are dependent on location. Mol Imaging Biol.

[51] Saha D, Watkins L, Yin Y, Thorpe P, Story MD, Song K (2010). An orthotopic lung tumor model for image-guided microirradiation in rats. Radiat Res.

[52] Zhang Z, Wodzak M, Belzile O, Zhou H, Sishc B, Yan H (2016). Effective rat lung tumor model for stereotactic body radiation therapy. Radiat Res.

