# Supplementary material for: Anti-tumor efficacy of CKD-516 in combination with radiation in xenograft mouse model of lung squamous cell carcinoma
Source: BMC Cancer. 2020 Nov 3;20:1057. doi: 10.1186/s12885-020-07566-x (PMC7607852; doi:10.1186/s12885-020-07566-x)
Supplement: Supplementary file 1 — Additional file 1. Short-term and long-term drug administration schedules. [file 12885_2020_7566_MOESM1_ESM.pdf]

Table S1. Short-term and long-term drug administration schedules.

Groups	Treatment Schedules	
	Short-term treatment<br>(1 cycle)	Long-term treatment<br>(3 cycles)
Vehicle	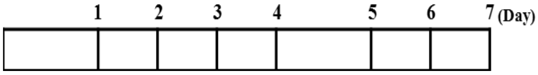	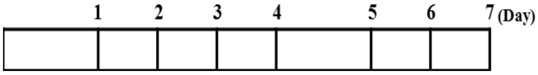
IR	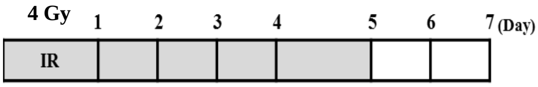	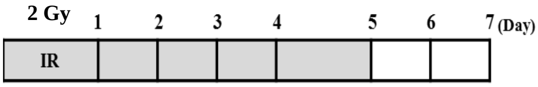
CKD-516	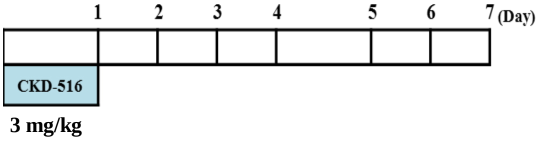	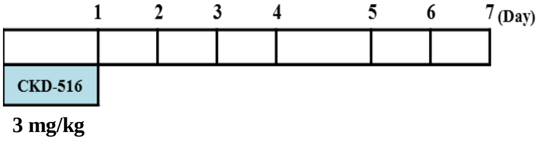
CKD-516<br>+ IR (d1)	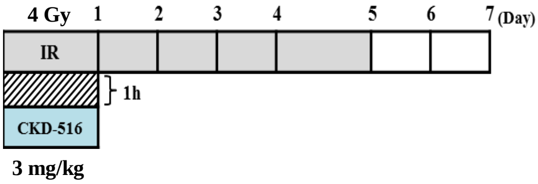	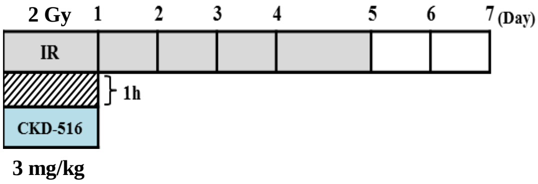
CKD-516<br>+ IR (d1, 5)	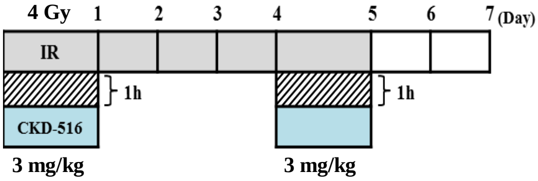	
