# Supplementary material for: Anti-tumor efficacy of CKD-516 in combination with radiation in xenograft mouse model of lung squamous cell carcinoma
Source: BMC Cancer. 2020 Nov 3;20:1057. doi: 10.1186/s12885-020-07566-x (PMC7607852; doi:10.1186/s12885-020-07566-x)
Supplement: Supplementary file 2 — Additional file 2. Toxicity of low- or high-dose IR in BALB/c nude mice. (A) The shielding device was made of 4 mm thick lead. The anesthetized mice were fixed in a 50 mL tube and irradiated with a lead shield. (B) Tumor growth and (C) body weight according to radiation dose. (D) Tumor growth during long-term treatment with 4 Gy of IR. [file 12885_2020_7566_MOESM2_ESM.pdf]

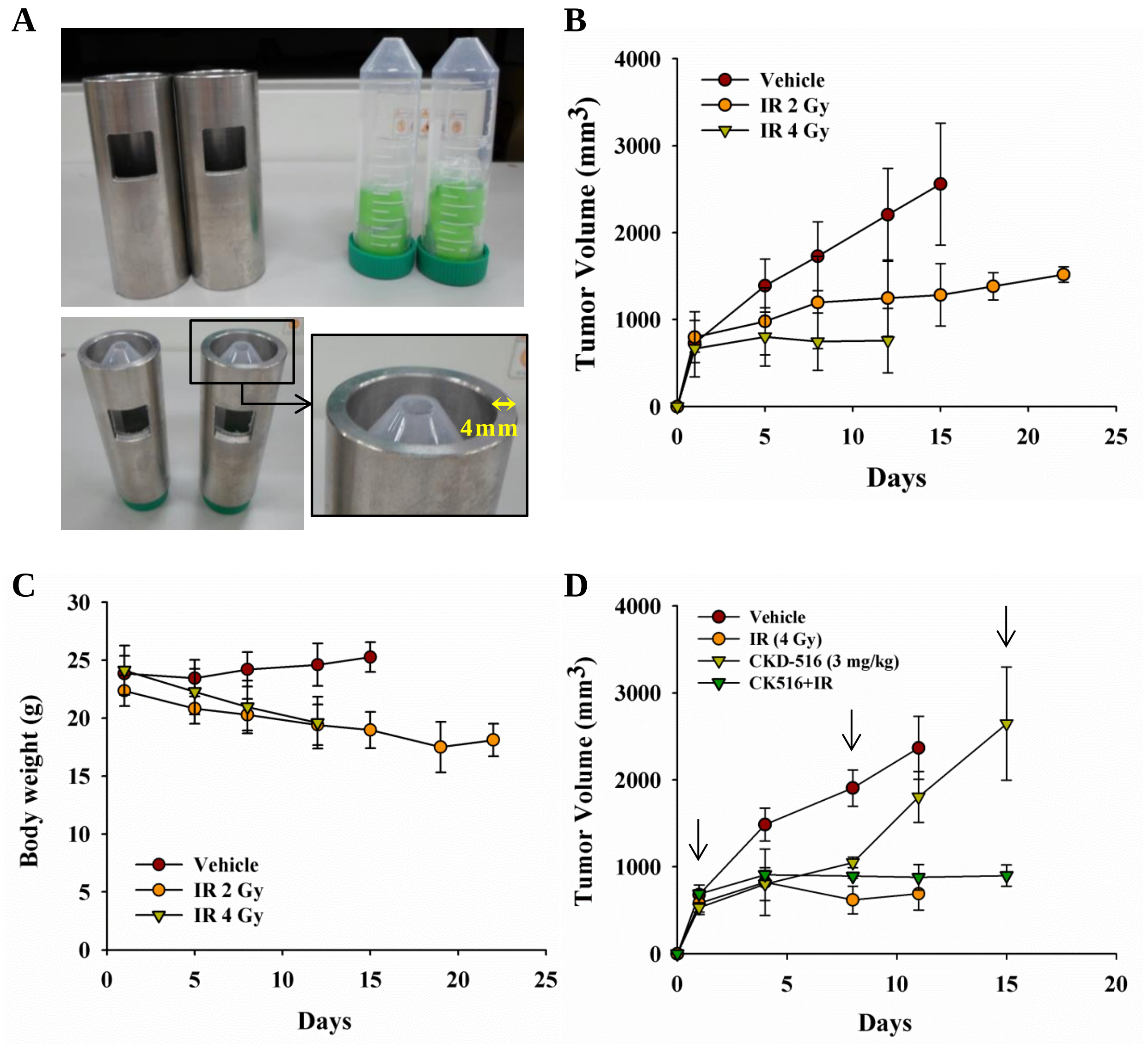

Additional file 2. Toxicity of low or high dose irradiation in BALB/c nude mice. (A) The shielding device was made of 4 mm thick lead. The anesthetized mice were fixed in a 50 mL tube, and then irradiated with a lead shield. Tumor growth (B) and body weight (C) according to radiation dose. (D) Tumor growth during long-term treatment with 4 Gy irradiation.
