# Supplementary material for: Anti-tumor efficacy of CKD-516 in combination with radiation in xenograft mouse model of lung squamous cell carcinoma
Source: BMC Cancer. 2020 Nov 3;20:1057. doi: 10.1186/s12885-020-07566-x (PMC7607852; doi:10.1186/s12885-020-07566-x)
Supplement: Supplementary file 3 — Additional file 3. IHC staining with HIF-1α, VEGF, Glut-1, and Ki-67 antibodies in all five treatment groups (72 h after the end of treatment). Mice were divided into five groups according to the dosing and treatment schedule: vehicle (PBS), IR alone (4 Gy/day), CKD-516 alone (3 mg/kg), and CKD-516 (3 mg/kg, day 1 or days 1 and 5) combined with IR.Scale bar: 200 μm. Magnification: × 100. [file 12885_2020_7566_MOESM3_ESM.pdf]

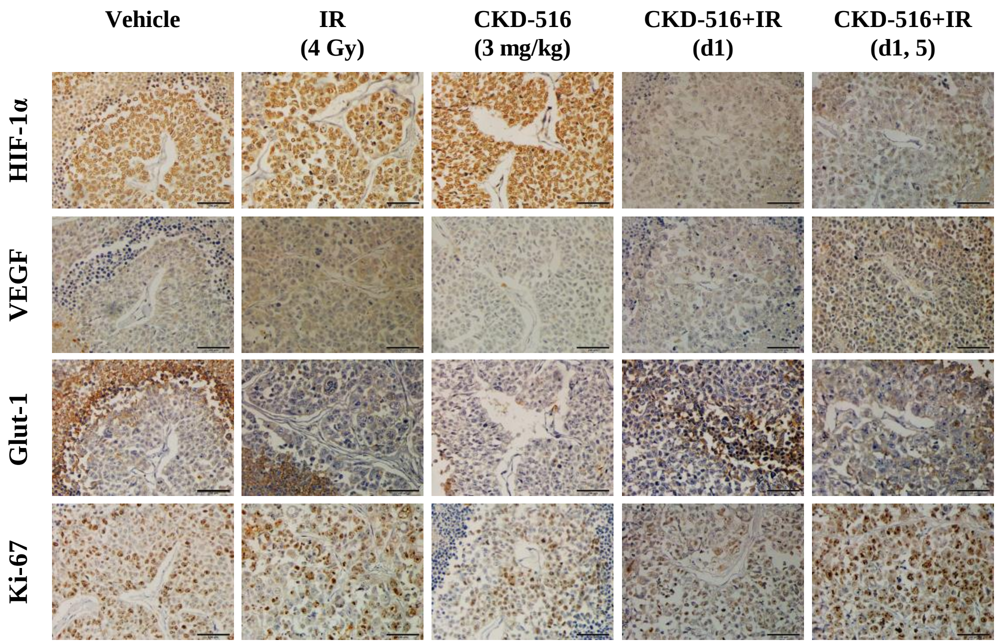

Additional file 3. IHC staining with HIF-1α, VEGF, Glut-1, and Ki-67 antibodies in all five treatment groups (72 h after the end of treatment). Mice were divided into five groups according to the dosing and treatment schedule: vehicle (PBS), IR alone (4 Gy/day), CKD-516 alone (3 mg/kg), and CKD-516 (3 mg/kg, day 1 or days 1 and 5) combined with IR.Scale bar: 200 μm. Magnification: ×100.
